# Regenerative Potential of PDL‐Derived Small Extracellular Vesicles

**DOI:** 10.1111/jre.13356

**Published:** 2024-11-24

**Authors:** Anahid A. Birjandi, Steven Lynham, Eva Matalova, Mandeep Ghuman, Paul Sharpe

**Affiliations:** ^1^ Centre for Craniofacial and Regenerative Biology, Faculty of Dentistry Oral & Craniofacial Sciences, Kings College London London UK; ^2^ Centre of Excellence for Mass Spectrometry Denmark Hill, King's College London London UK; ^3^ Institute of Animal Physiology and Genetics Czech Academy of Sciences Brno Czech Republic; ^4^ Centre for Host‐Microbiome Interactions. Faculty of Dentistry, Oral & Craniofacial Sciences King's College London London UK

## Abstract

Treatment of gingival fibroblasts with PDL extracellular vesicles results in promotion of Wnt signalling pathway and osteogenic differentiation. PDL secretome shows selective wound healing and matrix remodelling which can have implications for future periodontal regenerative strategies.

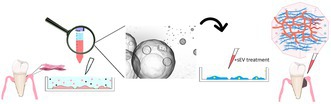

## 1 Introduction

The optimal healing outcome following periodontal treatment is complete periodontal regeneration which is very rarely achieved. Periodontal ligament (PDL) cells promote direct connective tissue attachment consistent with regenerative outcomes, whereas gingival connective tissue is not innately permissive. An alternative approach to promote regeneration is to alter the potential of gingival connective tissue to promote bone formation and connective tissue attachment. Multipotent mesenchymal stem cells have been isolated from gingival tissues and promote peri‐implant bone regeneration [1].

With the significance of paracrine signalling in tissue regeneration, there has been a focus on the therapeutic potential of secreted extracellular vesicles (sEV). sEV are cell‐derived particles that carry biological cargo and are naturally released by almost all cells demonstrating therapeutic and regenerative properties in various tissues [2]. Paracrine signals from isolated PDL cells have previously been shown to induce expression of PDL and bone markers in gingival cells suggesting that these cells are amenable to pro‐generative phenotypic change [3]. We investigated the effect of human PDL cell‐derived sEV on gingival fibroblasts. Furthermore, sEV were characterised and compared to those from a non‐oral source to identify candidates that may be selectively involved in oral wound healing.

## 2 Materials and Methods

Detailed methods for Cell culture, purification and characterisation of small extracellular vesicles, QPCR and label‐free proteomics are described in Appendix S1.

## 3 Results

### 3.1 sEV Purified From hPDL Induce Axin2 Expression and Promote Osteogenic Gene Expression in Gingival Fibroblasts

**FIGURE 1:**
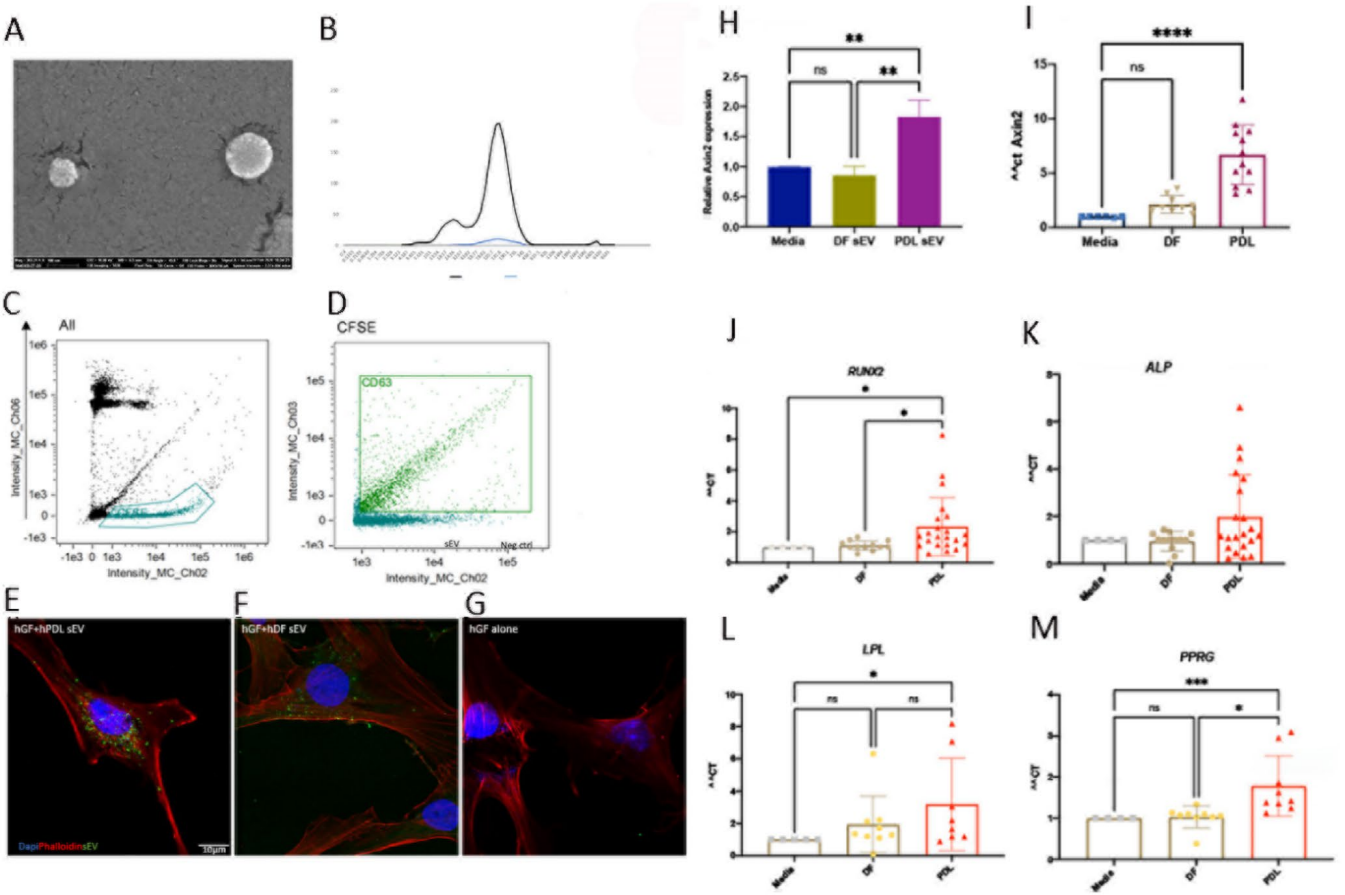
Treatment of gingival fibroblasts with sEV purified from PDL and DF. Purified sEV were characterised using SEM (A), DLS (B) and advanced flow cytometry showing CFSE + particles (C) and the proportion of CFSE+ particles that are also CD63+ (D). Confocal image of sEV derived from hPDL and hDF, stained with CFSE and taken up by gingival fibroblasts after 24 h treatment (E–G). Twenty‐four‐hour treatment of PDL‐sEV induced a higher level of *Axin2* expression in hGF in comparison to hDF‐sEV (H). Five‐day treatment of hGF results in significant upregulation of *Axin2* with PDL‐sEV treatment. Each data point corresponds to one batch of purified sEV (I). Gingival fibroblasts were treated with sEV derived from PDL and DF after reaching confluency for 10 days. Relative expressions of differentiation markers, osteogenic (J, K) and adipogenic (L, M). Each data point corresponds to one batch of purified sEV in all graphs.

Small extracellular vesicles were purified from human PDL (PDL‐sEV) and dermal fibroblasts (DF‐sEV). Scanning electron microscopy and dynamic light scattering demonstrate that these sEV are around 100–150 μm (Figure 1A,B). Expression of the pan‐exosome marker (CFSE) and an sEV‐specific marker, tetraspanin CD63 in CFSE+ particles by advanced flow cytometry confirmed that isolated particles contained small extracellular vesicles (Figure 1C,D).

Human gingival fibroblasts were treated with PDL‐sEV and DF‐sEV. Both sources of sEV demonstrated effective take‐up by gingival fibroblasts after 24 h (Figure 1E–G). Wnt signalling is crucial in the development and regeneration of periodontal tissue. Treatment of human gingival fibroblasts with PDL‐sEV resulted in induction of Wnt signalling via upregulation of *Axin2* expression, a downstream cytoplasmic target. Longer treatment of 5 days resulted in significant upregulation of *Axin2* gene expression in gingival fibroblasts treated with hPDL‐sEV in comparison to hDF‐sEV (Figure 1H,I). Additionally, a 10‐day treatment with PDL‐sEV resulted in significantly greater expression of a key marker for osteogenic differentiation, *RUNX* and *ALP* in most batches of purified vesicles tested (Figure 1J,K). Induction of adipogenic differentiation markers was more selective with a high level of *LPL* associated with both sEV types and significantly greater expression of *PPARG* in nearly all batches of PDL‐sEV (Figure 1L,M). With batch variation inherent in biological samples, not all markers were expressed at statistically significant different levels. Although overall a pattern of increased expression of differentiation markers across various batches of PDL‐sEV in comparison to DF‐sEV was observed.

### 3.2 PDL‐sEV Proteome Demonstrate Selective Wound Healing and Matrix Remodelling Signatures

**FIGURE 2:**
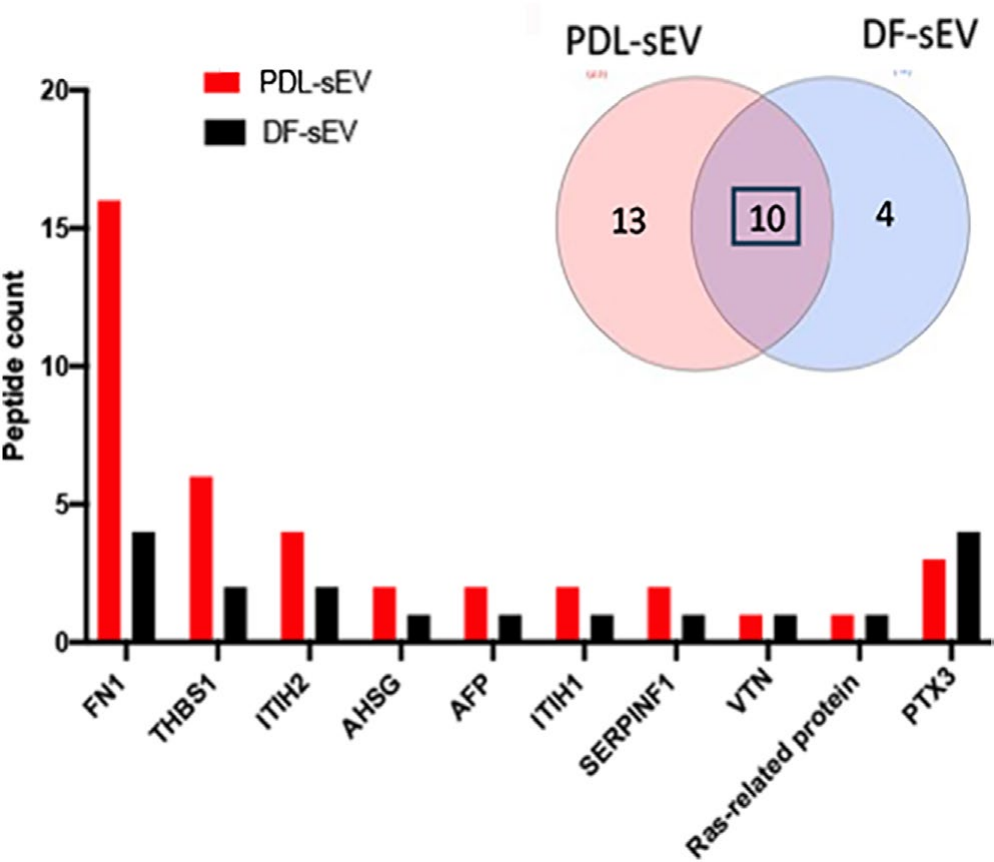
Proteome of sEV derived from PDL and DF. Venn diagram shows the number of shared proteins between PDL‐sEV and DF‐sEV and the bar graph demonstrates peptide count of these shared proteins.

Protein content of PDL‐sEV and DF‐sEV was investigated using label‐free proteomics (Data not shown). Data on peptide spectral matches (PSM) revealed 13 proteins unique to PDL‐sEV and 10 shared with DF‐sEV. GO enrichment analysis demonstrated that these proteins were enriched in haemostasis, regulation of coagulation, wound healing, platelet activation, signalling and aggregation (Figure S1). Most of the proteins shared between DF‐sEV and PDL‐sEV had higher peptide counts in PDL‐sEV such as fibronectin, thrombospondin, inter‐alpha‐trypsin inhibitor heavy and alpha antiplasmin (Figure 2).

## 4 Discussion

Given their therapeutic potential, sEV could be considered an attractive alternative to cell‐based treatments of periodontitis. Previous studies have shown the regenerative potential of sEV on periodontal tissue via angiogenesis, immunomodulation or chemotaxis [4, 5]. The results of this study suggest the importance of the role of angiogenesis. The results align with a previous study showing PDL cells can promote the osteogenic capacity of gingival cells via paracrine signalling. Furthermore, PDL‐EVs can promote proliferation, differentiation and migration of osteoblastic cells through MEK/ERK signalling [6, 7]. Our work expands this in comparing directly with DF‐EVs highlighting a selective signature of angiogenesis, wound healing and ECM rearrangement in PDL‐sEV.

Proteomic analysis showed a selectively higher total unique peptide count of fibronectin in PDL‐sEV and a signature of angiogenesis, wound healing and ECM rearrangement compared to dermal sEV. Fibronectin has multiple roles in tissue organisation and healing and increased expression in oral wounds [8]. PDL‐sEV also demonstrated high levels of prothrombin. Interestingly, elevated levels of plasminogen activator inhibitor‐1 (PAI‐1) activity are observed in periodontitis which results in impaired fibrinolysis and subsequently a prothrombotic state [9]. The differences in results between PDL and dermal cells potentially reflect differential wound healing capacity. Distinct patterns of angiogenesis and phenotypic differences between oral and dermal fibroblasts are known to contribute to dissimilar healing processes in these tissues [10]. In this study, we highlight the differences between the periodontal and dermal secretome potentially reflecting oral and dermal wound‐healing capacity. Further investigation of these differences is necessary for better understanding and assessment of their role as a potential therapy in enhancing periodontal regeneration.

## 5 Conclusion

PDL secretome demonstrates regenerative potential which can be harvested for periodontal regenerative strategies.

## Author Contributions

A.A.B. designed the study, conducted the experiments, created manuscript draft and critically revised it. P.S. and M.G. designed the study and critically revised the manuscript. S.L,. conducted proteomic experiment, performed mass spectroscopy and initial data analysis. E.M. revised the manuscript.

## Conflicts of Interest

The authors declare no conflicts of interest.

## Supporting information


**Figure S1.** Proteome of sEV derived from PDL.

Appendix S1.

## Data Availability

The data that support the findings of this study are available from the corresponding author upon reasonable request.
