# Supplementary material for: Regenerative Potential of PDL‐Derived Small Extracellular Vesicles
Source: J Periodontal Res. 2024 Nov 24;60(4):392–4. doi: 10.1111/jre.13356 (PMC12139699; doi:10.1111/jre.13356)
Supplement: Supplementary file 1 — Figure S1. Proteome of sEV derived from PDL. [file JRE-60-392-s002.docx]

Supplementary Figure


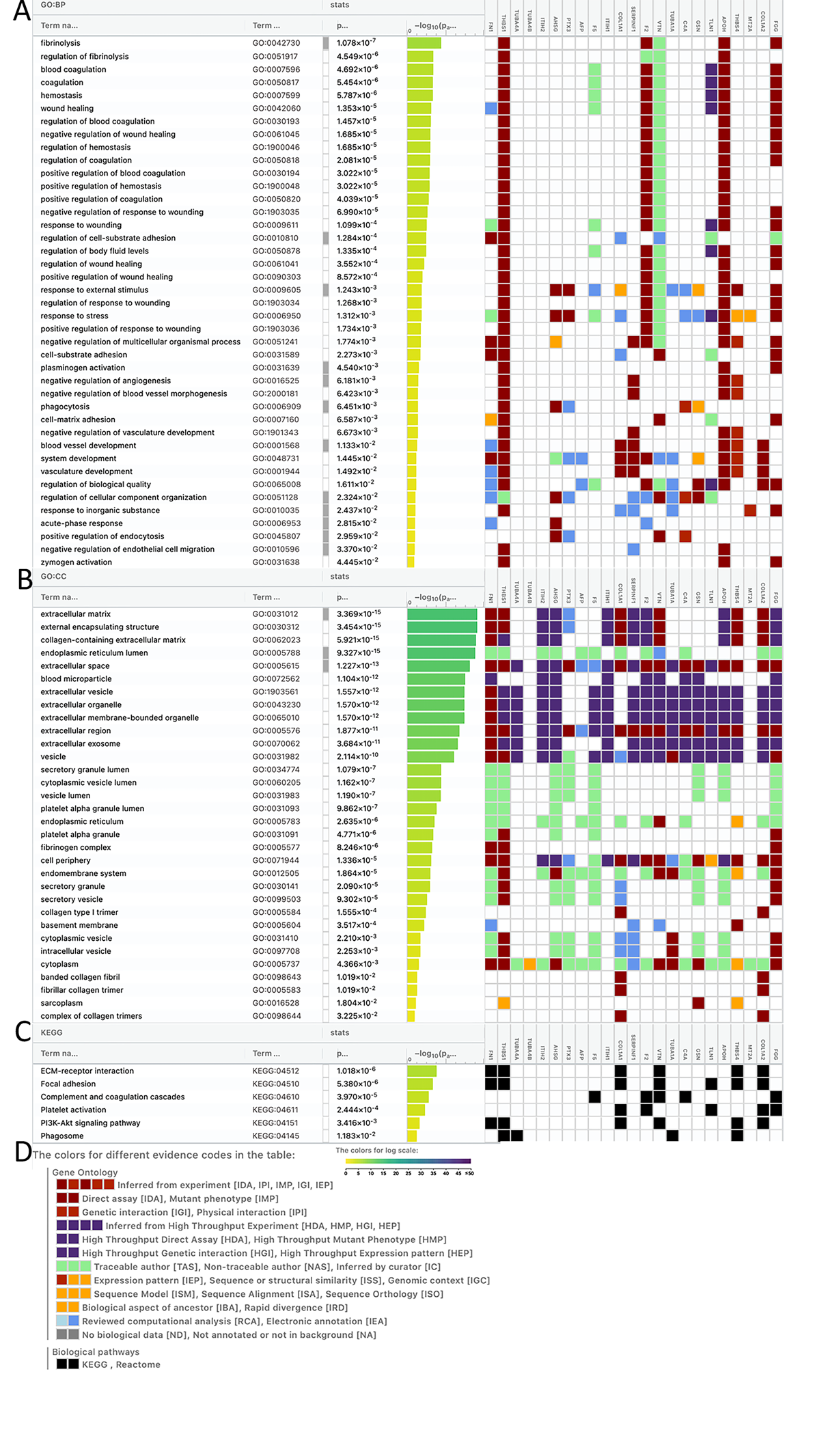


**Supplementary figure. Proteome of sEV derived from PDL.**GO enrichment analysis by g: profiler of all proteins identified in PDL- sEV and their associated biological process(A), cellular component (B), KEGG pathway (C) and Legends (D).
