# Supplementary material for: Regenerative Potential of PDL‐Derived Small Extracellular Vesicles
Source: J Periodontal Res. 2024 Nov 24;60(4):392–4. doi: 10.1111/jre.13356 (PMC12139699; doi:10.1111/jre.13356)
Supplement: Supplementary file 2 — Appendix S1. [file JRE-60-392-s001.docx]

**Methods**

**Cell culture**

Primary human PDL cells (hPDL) were a kind donation from Dr Ana Angelova, King’s College London. Human Dermal Fibroblasts from the axillary region of a healthy 38 (F) year old, with no significant medical problems and no smoking history were a kind gift from Prof. I. Mackenzie, Queen Mary, University of London. Primary human gingival fibroblasts were established from tissues from healthy third molar teeth extracted from healthy patients aged 20 (F), 23(M) and 35 (F) years old, with no significant medical problems and no smoking history. All patients were attending a surgical appointment at the Department of Oral Surgery, Guy's Hospital. Kent NHS Research Ethics Committee approved the protocol (Reference No: 11/LO/0259), and informed written consent was obtained from all the subjects.

Primary cells were used at passage 2-3. Cells were expanded in DMEM high glucose for optimum growth, 10% exosome depleted FBS (Thermofisher, A2720803), 1% L-Glutamine and 1% Penicillin/Streptomycin. Our in-house pilot experiments demonstrated optimum growth of PDL, GF and DF cells in high glucose DMEM media.

For differentiation assays, Gingival fibroblast cells were seeded in 24 well plates. Once reaching confluency, gingival fibroblasts were treated with sEV derived from PDL or DF for 10 days and then assessed for makers of osteogenic and adipogenic differentiation.

**Purification and characterization of small extracellular vesicles**

Small extracellular vesicles (sEV) were purified from cells in complete media DMEM 10% exososome depleted FBS (Thermofisher, A2720803). Differential centrifugation at 300g 10 min, 2000g 10 min, 10000g for 1 hr and 3 hr ultracentrifugation at 100,000g were used for the purification of sEV. Purified sEV were resuspended in endotoxin free PBS and stored in -80 till further use. For every experiment, 3 batches of purified exosomes ( biological replicates) and 3 technical replicates were used . Media only samples were used as our control and subject to all the experiments in order to eliminate factors associated with the media ie proteins identified in the media .

For scanning electron microscopy (SEM), sEV were fixed in 4% paraformaldehyde (PFA). Particles were spotted on a clean flat silicon surface from isopropanol suspension in a 10 µL volume. The particles were dried and imaged by in-lens detector in a Karl Zeiss XB1540 SEM, with voltage 10 keV. (Thermo Scientific Inc.)

For dynamic light scattering (DLS) , sEV suspended in endotoxin free PBS were transferred into sterile cuvettes. PBS was adopted as the dispersing solvent. All analyses were carried out at 25°C, and each purification had three replicates. All measurements were carried out 12 times per sample. DLS data were analysed using Zetasizer software (V 7.11) (Malvern Panalytical, Malvern, United Kingdom).

Aminis Advanced nano flow cytometry was used to detect labelled sEV suspended in endotoxin free PBS. Briefly, purified sEV were incubated with pan exosome marker CFSE and tetraspanin CD63 (mAbs; BioLegend, San Diego, CA, USA) which is another marker of sEV for 30 min and a subject to Amnis image stream. Multiple controls were used for this imaging such as endotoxin free PBS only, CD63 only and CFSE only.

**I**mages of sEV uptake by PDL and DF were obtained using confocal microscopy with LSM980 Zeiss using Airyscan. For all experiments, DF-sEV and PDL-sEV were coded alphabetically so the performer was blind to the groups being used in each experiment.

**Quantitative PCR**

Human gingival fibroblasts were treated with PDL-sEV and compared to those treated with extracellular vesicles derived from dermal fibroblasts (DF-sEV), a control to exclude any effect attributed to general EV treatment. For Axin2 expression, gingival fibroblasts were plated in triplicates in 24-well plates and incubated at (37°C, 5% CO2/95% air, 100% humidity) for 24h using standard culture medium containing EV specific FBS (Endotoxin free FBS Thermofisher, A2720803). A volume corresponding to 100µg protein of PDL-sEV and DF-sEV were used for treatment of gingival fibroblasts for 24 hours. In all assays various batches of purified sEV were used. Each data point in qPCR graphs corresponds to a batch of purified sEV. For differentiation assays , gingival fibroblast cells were seeded in 24 well plates in normal media.  Once reaching confluency, gingival fibroblasts were treated with sEV derived from PDL or DF in normal media for 10 days and then assessed for makers of osteogenic and adipogenic differentiation.

Once the media was removed, and cells were lysed with Trizol for extraction of RNA and real time qPCR as recommended by the manufacturer’s instructions. RNA was reverse transcribed using random primers (M-MLV Reverse Transcriptase kit, Promega, Madison, WI, USA) according to the manufacturer’s instructions. Gene expression was then assayed by real-time qPCR using Sybr Green (Roche, Basel, Switzerland) on a Rotor-Gene Q cycler (Qiagen, Hilden, Germany) system. Beta-actin was used as the reference gene (Forward-GGCTGTATTCCCCTCCATCG, Reverse-CCAGTTGGTAACAATGCCTGT) and Axin2 expression levels was the read-out for Wnt pathway activity (Forward-TGACTCTCCTTCCAGATCCCA, Reverse-TGCCCACACTAGGCTGACA), For osteogenic differentiation, Runx2 (Forward-TGGTTACTGTCATGGCGGGTA, Reverse-TCTCAGATCGTTGAACCTTGCTA), ALP (Forward ACCACCACGAGAGTGAACCA, Reverse-CGTTGTCTGAGTACCAGTCCC), for chondrogenic differentiation Sox9 (forward- AGCGAACGCACATCAAGAC, Reverse-CTGTAGGCGATCTGTTGGGG). For adipogenic differentiation: LPL (Forward- TCATTCCCGGAGTAGCAGAGT, Reverse- GCCACAAGTTTTGGCACC); PPARG (Forward- ACCAAAGTGCAATCAAAGTGGA, Reverse- ATGAGGGAGTTGGAAGGCTCT). Reactions were performed in triplicate and relative changes to the reference gene were calculated by the 2−∆∆CT method where CT is the threshold cycle. Normal distribution of results was tested with Shapiro-Wilk test. Statistical significance was reported using one-way ANOVA and Tukey multiple comparisons using GraphPad Prism 9. For comparison of differentiation marker expression, the Kruskal Wallis test was used. Adjusted P values reported in graphs according to New England Journal of Medicine guidelines: P < 0.001 (***), P < 0.002 (**), P < 0.033 (*), P > 0.12 (ns).

**Proteomics**

Protein content of PDL-sEV and DF-sEV was investigated using label free proteomics. Since these cells were cultured in media containing exosome depleted FBS, the medium alone was used as a control and subjected it to all steps of sEV purification and proteomics.

Small extracellular vesicles were purified from periodontal ligament cells and dermal fibroblasts as mentioned before. Each submitted sEV sample correspond to a pool of 3 batches of purified exosomes within each group. Control samples of media-only was subjected to ultracentrifugation and resuspended in exosome depleted PBS and treated similar to experimental groups. Samples were loaded into precast Tris-Bis gel for short resolution to ‘stack’ the protein complement and purify the sample for contaminants prior to protein digestion. All samples were resolved for 20 minutes and stained overnight in a colloidal protein stain at RT. Following incubation, gels were de-stained with ddH2O to compare intensity of protein loading was consistent with protein estimation calculations. Enzymatic digestion and peptide extraction were performed according to protocol.

*LC-MS/MS*

The peptide sample was resuspended in 36ml of resuspension buffer (2% acetonitrile in 0.05% formic acid), 6ml of which was injected to be analysed by LC-MS/MS. Chromatographic separation was performed using a U3000 UHPLC NanoLC system (ThermoFisher Scientific, UK). Peptides were resolved by reversed phase chromatography on a 75mm C18 Pepmap column (50cm length) using a three-step linear gradient of 80% acetonitrile in 0.1% formic acid. The gradient was delivered to elute the peptides at a flow rate of 250nl/min over 60 min starting at 5% B (0-5 minutes) and increasing solvent to 40% B (5-40 minutes) prior to a wash step at 99% B (40-45 minutes) followed by an equilibration step at 5% B (45-60 minutes). The eluate was ionised by electrospray ionisation using an Orbitrap Fusion Lumos (ThermoFisher Scientific, UK) operating under Xcalibur v4.3. The instrument was first programmed to acquire using an Orbitrap-Ion Trap method by defining a 3s cycle time between a full MS scan and MS/MS fragmentation by collision induced dissociation. Orbitrap spectra (FTMS1) were collected at a resolution of 120,000 over a scan range of m/z 375-1600 with an automatic gain control (AGC) setting of 4.0e5 (100%) with a maximum injection time of 35 ms. Monoisotopic precursor ions were filtered using charge state (+2 to +7) with an intensity threshold set between 5.0e3 to 1.0e20 and a dynamic exclusion window of 35s ± 10 ppm. MS2 precursor ions were isolated in the quadrupole set to a mass width filter of 1.6 m/z. Ion trap fragmentation spectra (ITMS2) were collected with an AGC target setting of 1.0e4 (100%) with a maximum injection time of 35 ms with CID collision energy set at 35%.

For data base searching, raw mass spectrometry data from injections was processed into peak list files using Proteome Discoverer (ThermoScientific; v2.5) in a label-free quantitative workflow. Raw data was processed and searched using the Mascot search algorithm (v2.6.0; [www.matrixscience.com](http://www.matrixscience.com/)) and the Sequest search algorithm (Eng *et al*; PMID 24226387) against the Uniprot human Taxonomy database. The reporter ion intensity values (absolute area under the peak) for each peptide spectral match are grouped with other identified peptides and calculated at the protein level identification as a grouped abundance. Spectrum selector was set at minimum of 350 Da and maximum of 1000 Dalton precursor mass and S/N threshold of 1.5. FDR value was set at 0.01. For analysis, Proteome Scaffold 5 software was used. After filtering entries shared with media control, experiment-wide grouping with protein cluster with peptide threshold of 95% minimum was performed. For functional enrichment analysis G profiler was used with significance threshold set at Bonferroni correction Term size set at 50 (18,19). MS/MS analysis identified 114 proteins in 91 clusters across sEV  samples and media only  with protein threshold of 99% and peptide threshold 95% ( data not shown) . After filtering entries with values in media control such as keratin and albumin, 27 proteins were identified to be unique to sEVs and the distribution of unique proteins in sEV derived from PDL and DF was analysed. Raw data will be deposited to the requested portal if required.
